# A model of traumatic brain injury in rats is influenced by neuroprotection of diurnal variation which improves motor behavior and histopathology in white matter myelin

**DOI:** 10.1016/j.heliyon.2023.e16088

**Published:** 2023-05-09

**Authors:** R.J. Martínez-Tapia, F. Estrada-Rojo, T.G. López-Aceves, S. García-Velasco, V. Rodríguez-Mata, E. Pulido-Camarillo, A. Pérez-Torres, E.Y. López-Flores, P. Ugalde-Muñiz, R. Noriega-Navarro, L. Navarro

**Affiliations:** a Laboratory of Neuroendocrinology, Departamento de Fisiología, Facultad de Medicina, Universidad Nacional Autónoma de México, México City, Mexico; b Programa Regional de Posgrado en Biotecnología, Facultad de Ciencias Químico-Biológicas, Universidad Autónoma de Sinaloa, Culiacán, Sinaloa, Mexico; c Departamento de Biología Celular y Tisular, Facultad de Medicina, Universidad Nacional Autónoma de México, Mexico City, Mexico; d Residente de Anatomía Patológica, CMN “20 de Noviembre”, ISSSTE, Ciudad de México, Mexico

**Keywords:** Traumatic brain injury, Diurnal variation, White matter, Myelin, Neuroprotection

## Abstract

Traumatic brain injury (TBI) represents a significant public health concern and has been associated with high rates of morbidity and mortality. TBI generates two types of brain damage: primary and secondary. Secondary damage originates a series of pathophysiological processes, which include metabolic crisis, excitotoxicity, and neuroinflammation, which have deleterious consequences for neuronal function. However, neuroprotective mechanisms are also activated. The balance among these tissue responses, and its variations throughout the day determines the fate of the damage tissue. We have demonstrated less behavioral and morphological damage when a rat model of TBI was induced during the light hours of the day.

Moreover, here we show that rats subjected to TBI in the dark lost less body weight than those subjected to TBI in the light, despite no change in food intake. Besides, the rats subjected to TBI in the dark had better performance in the beam walking test and presented less histological damage in the corpus callosum and the cingulum bundle, as shown by the Klüver-Barrera staining.

Our results suggest that the time of day when the injury occurs is important. Thus, this data should be used to evaluate the pathophysiological processes of TBI events and develop better therapies.

## 1 Introduction

Traumatic brain injury (TBI) is a serious public health problem. The most recent Global Burden Disease (GBD) report established that in 2016, there were 27.08 million new cases of TBI in the five World Health Organization (WHO) regions. This means a prevalence of 369 per 100 000 [1]. TBI is defined as any alteration of brain function or other evidence of brain pathology that is caused by an external force [2], i.e., bump, blow, or jolt to the head or a penetrating head injury [3].

The study of TBI is a considerable challenge due to the complexity of the activated pathophysiological pathways. However, two types of damage have been described after TBI: primary damage that occurs at the moment of impact and is irreversible, and around this primary damage, secondary damage that occurs in the healthy tissue as a result of the pathophysiological cascades that are activated, which involves a series of molecular and cellular pathways that aggravate the damage over time [4]. These pathways involve, among others, excitotoxicity damage, mediated mainly by the *N*-methyl-d-aspartate (NMDA) receptor [5], oxidative stress damage through the production of reactive oxygen and nitrogen species [6], and neuroinflammation, in which microglial cells and astrocytes play a key role [7].

The damage caused by TBI differs in its effects on the gray matter (which contains neural cell bodies, dendrites, glia, synapses, and capillaries) and the white matter (which is primarily made up of interconnected myelinated axon tracts and in which oligodendrocytes are typically arranged in linear rows between the nerve fibers), which is one of the aspects to emphasize [8,9]. This disparity in damage has been related to differential movement between gray and white matter during acceleration-deceleration movement, which results in disaggregation between the two materials and diffuse axonal injury (DAI) [9]. In addition, when cognitive, behavioral, and sensorimotor dysfunctions appear more than a year after a TBI, this damage has been linked to them [10].

We previously demonstrated that the severity of damage in TBI depends on the time of day it was induced. Our findings indicate that subjects with TBI induced at night present better neurobehavioral scale scores [11,12], and we recently found a less histopathological changes of the gray matter in the secondary damage zone than subjects with TBI induced during the day [13]. However, there is still little evidence regarding whether white matter preservation, specifically the myelin and oligodendrocytes, in the central nervous system (CNS) in the acute period following TBI are influenced by diurnal variation and whether it shows less damage when induced at night. In this work, we analyzed the effects of TBI induced at two different points of the light-dark cycle, using a motor test and correlated the results with a histopathological evaluation of the white matter perilesional to the TBI.

## 2 Materials and Methods

### 2.1 Animals

Male Wistar rats weighing 250–300 g (10–12 weeks old) were housed individually and habituated to the environmental conditions 15 d prior to the experiments in a room with a temperature range of 21±2 °C and food and water *ad libitum*. Additionally, we established a light:dark cycle of 12:12 h (with lights on at 8:00 a.m., and lights off at 8:00 p.m.). All tests followed the requirements of the Ethics Committee of the Faculty of Medicine at the National Autonomous University of Mexico (UNAM) (project 018/2016; authorized on April 5, 2016), the Care and Use of Animals, and Official Mexican Regulations (NOM062-ZOO-1999).

#### 2.1.1 Experimental design

**Fig. 1:**
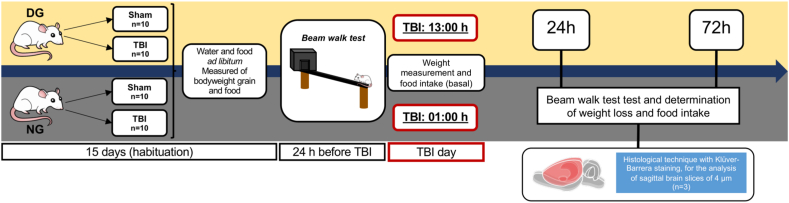
Flow chart of experimental design used.

The experimental subjects were divided into two groups: the day group (DG) and the night group (NG). TBI was induced in DG at 13:00 h (5 h after turning on the lights); for the NG, TBI was induced at 01:00 h (5 h after lights out). In both groups, the behavioral test was performed 24 h before TBI and repeated at 24 and 72 h after TBI occurred (Fig. 1). The test for the NG was conducted in a dark room with red light <10 lx to avoid disrupting the sleep-wake cycle [14,15]. Finally, both the DG and NG were subdivided into Sham (n = 10) and TBI (n = 10) subgroups, with the Sham group consisting of manipulation and anesthesia without the TBI induction.

##### 2.1.1.1 Body weight and food intake

Food intake and body weight were measured 24 h before TBI, and 24, 48, and 72 h after TBI. For both, food intake and body weight, we calculated the difference (Δ). Body weight was measured using triple beam balance scale (Ohaus Triple Beam Scale 700 Series®). Food intake was determined using an analytical balance (A&D Instruments, HM-120®).

#### 2.1.2 TBI model

TBI was induced using a closed head injury model (adapted from Shohami et al., 1988). It belongs to the models that use gravitational force to produce lesions in the skull and whose use has been reported for several years [[16], [17], [18], [19]]. We anesthetized our experimental subjects before inducing TBI. Rats were sedated with 4.0% isoflurane (Sofloran®Vet, PiSA Agropecuaria, Hgo., Mexico) in an acrylic gas-anesthesia chamber (25 cm × 11 cm × 10 cm) supplemented with O_2_. Once the righting reflex was confirmed to be absent, each rat was placed in a stereotaxic system with a nasal cone, through which the O_2_/isoflurane mixture continued to be supplied. The isoflurane level was adjusted to 3.0% to maintain an adequate anesthetic depth during surgery. The rat head was disinfected with chlorhexidine, and the skull was exposed through a midline skin incision. At coordinates P = −2 and L = 1.4, the stereotaxic device was used to locate the site of the TBI (primary motor cortex) [20]. A pneumatic piston calibrated at 40 psi pressure and 6 mm depth was used to induce the TBI. Isoflurane delivery was stopped once TBI was completed, and the rats were returned to their cage. Before recovering the righting reflex, the rat was placed in a supine position. The rats were then returned to the vivarium.

##### 2.1.2.1 Beam walk test

A beam walk test was used to assess the coordination and, the fine motor skills of the rats (described in and adapted from previous works, [[21], [22], [23]]). The test was used for both the DG and NG, and the Sham and TBI subgroups. We used a 100 × 2 cm square wooden bar elevated 40 cm above the table on wooden supports to perform the test. A 12 × 15 × 26 cm black escape box was placed at the opposite end with a 7 × 9 cm opening. For four consecutive days before the injury, the rats were placed at the free end and trained to cross the bar and enter the opposite end with the escape box. The time it took the animal to traverse the escape black box with its forelegs (latency) and the number of errors made (such as exceeding the maximum test time of 60 s or falling off the bar) were recorded. The baseline measurement was collected 24 h before TBI, and for statistical analysis, the average scores of three measurements were taken 1 h before euthanasia and 24, and 72 h after the trauma. The observer recorded the time it took the animal to cross the black escape box with its front paws.

### 2.2 Tissue processing and staining

At 24 and 72 h after TBI induction, rats (n = 3) were profoundly anesthetized with sodium pentobarbital (Pisabental®, 50 mg/kg i.p., PiSA Agropecuaria, Hgo., México). Then, the rats were whole-body perfused to introduce by a needle inserted into the left ventricle a sodium phosphate buffer followed by 4% buffered paraformaldehyde at room temperature, which exits by a previously cut made in the right atrium. We conducted the perfusion for about 30 min. Next, the brains were removed and maintained for at least 24 h in 4% buffered paraformaldehyde. After that, they were rinsed in tap water, dehydrated in ascending ethanol grades, cleared in xylene, and embedded in paraffin. Posteriorly, we obtained three series of 4 μm-thick sagittal sections spanning the midline to the left side of the brain (the TBI zone), and Klüver-Barrera (KB) stain method was used to evaluate the histopathological changes in white matter myelin (WM-M). The KB technique is a double Nissl staining using Cresyl Violet and Luxol Fast Blue staining, in which the myelin sheath stains in navy blue.

#### 2.2.1 Image acquisition

In the cingulum bundle (CB) and corpus callosum (CC), morphological alterations in myelin were observed and quantified. We obtained a total of 50 microscopic fields using a 40 × objective. Each area per field was previously calibrated at 19 700 μm^2^. Images were acquired using a CX31 Olympus microscope equipped with a digital camera Infinity1 (Teledyne Lumenera®), and the Infinity Analyze® software (version 6.3.0).

#### 2.2.2 Histopathological description and morphometric analysis

A medical pathologist qualitatively evaluated the changes in WM-M in the CB and CC for the DG and NG using a 400 × total magnification photomicrographs in a single-blind design. We employed a four-point scale that had been previously used to evaluate the damage of WM-M in Klüver-Barrera stain [[24], [25], [26]]. The criteria are established in detail in Table 1. The counts in the tissue sections were averaged. Additionally, FIJI software (v.2.0.0, https://imagej.net/Fiji) was used for cell counting and to determine the microscopic field areas.

**Table 1:** Criteria for the evaluation of myelin in white matter with Klüver-Barrera stain method.

Grade	Description
0	Normal
1	Disarrangement of the nerve fibers
2	Formation of marked vacuoles
3	Disappearance of myelinated fibers
Lie et al., 2013; Miyamoyo et al., 2004; Wakita et al., 1999.	

### 2.3 Statistical analysis

The results were reported as the mean ± standard error of the mean (SEM). GraphPad Prism software (GraphPad Software Inc., San Diego, CA, United States) was used for the statistical analyses. Unless otherwise indicated, the data met the assumptions of equal variances (Spearman's test for heteroscedasticity and homoscedasticity plot). To analyze the statistical differences in food intake, body weight, cylinder test, and WM-M counting, we used a two-way ANOVA and Tukey's multiple comparisons corrections. For neurobehavioral scores, Kruskal–Wallis and Mann–Whitney U tests were used. Differences were significant at *p* < 0.05.

## 3 Results

### 3.1 The main difference in food intake and body weight loss occurs 24 h after TBI

**Fig. 2:**
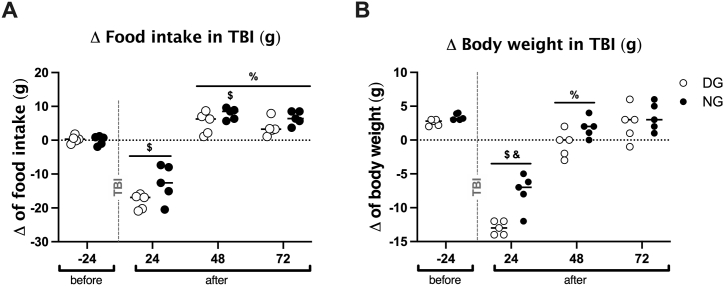
The time of day at which TBI is induced determines the change in food intake and body weight. Effect of inducing TBI at two different time points, in the day (white symbols) and at night (black symbols) on the difference (Δ) in food intake (A) and the Δ of body weight (B) in the TBI groups (n = 5 each subgroup). Data are expressed as mean ± SEM. Two-way ANOVA test and Tukey's *post-hoc* test. ^$^ different from basal (−24 h); ^%^ different from 24 h after TBI; ^&^ between DG and NG.

In the analysis of change in food intake for the DG and NG with TBI (Fig. 2A), we observed a significant influence of trauma between baseline intake (−24 h) and 24 h after TBI (*F*_3,32_ = 130.8, *p* < 0.0001). However, at 48 h and 72 h after the trauma, there was a statistically significant gain compared with the 24 h after TBI for both, the DG (*p* < 0.0001) and NG (*p* < 0.0001); also, at 48 h after the trauma, there was no significant difference compared with the baseline (−24 h) of the DG (*p* = 0.1528), whereas a significant difference for the NG (*p* = 0.0021). Interestingly, when comparing 48 h vs. 72 h after TBI, there were no longer any differences, either in the DG (*p* = 0.9947) or in the NG (*p* = 0.9961). Finally, although our statistical analysis determined that there was also an influence of the Day and Night groups (*F*_1,32_ = 9.261, *p* < 0.0001), the *post-hoc* analysis did not show differences between the Day and Night groups at - 24 h (*p* > 0.999), 24 h (*p* = 0.070), 48 h (*p* = 0.763), or 72 h (*p* = 0.743) after TBI.

Regarding the analysis of change in body weight (Fig. 2B), we observed an influence of TBI (*F*_3,32_ = 125.4, *p* < 0.0001) in both the DG (*p* < 0.0001) and the NG (*p* < 0.0001) at 24 h after trauma. At 48 h after TBI, recovery was observed for both, the Day group (*p* < 0.0001) and for the Night group (*p* < 0.0001), when compared with 24 h after TBI. This difference was maintained at 72 h after TBI, but there were no significant differences compared with the measurements 48 h post-TBI for both for the DG (*p* = 0.1763) and the NG (*p* = 0.8530). Finally, we compared the NG with the DG (*F*_1,32_ = 17.95, *p* = 0.0002) and found a significant difference only at 24 h after TBI, with a lower change in weight in the NG compared with that in the DG (*p* = 0.0010).

### 3.2 Beam walking test scores vary depending upon the time of TBI induction

**Fig. 3:**
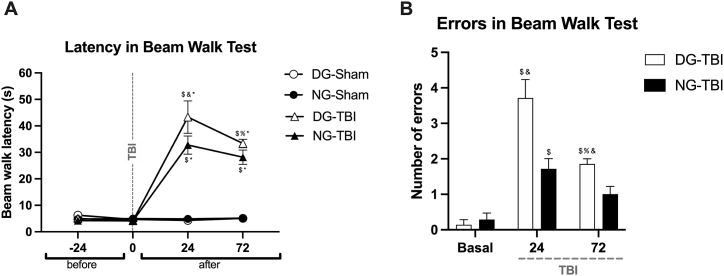
Analysis of the beam walking test according to the time of TBI induction. We observed an increase in the latency of DG and NG at 24 h and 72 h after TBI compared to Sham; however, NG had a statistically lower latency than DG at both 24 h and 72 h (A). Regarding errors, NG had significantly fewer errors than DG at both 24 and 72 h after TBI (B). Data are expressed as the mean ± SEM. (A) Two-way ANOVA and Tukey *post-hoc* test. ^$^ different from basal (−24 h); ^%^ different from 24 h after TBI; * different from Sham; ^&^ between DG and NG. (B) Kruskal–Wallis test and Mann–Whitney *U* test as *post-hoc*.

The beam walk test evaluated (Fig. 3) the loss of fine motor activity following TBI induction. The time it took to cross the bar to the escape box (latency) (Fig. 3A) was significantly influenced by TBI (*F*_3,72_ = 82.67, *p* < 0.0001). We found differences for the DG at 24 h (*p* < 0.0001) and at 72 h (*p* < 0.0001) and for the NG at 24 h (*p* < 0.0001) and 72 h (*p* < 0.0001) compared with the baseline (−24 h). Furthermore, we found differences between Sham and TBI at 24 h (*p* < 0.0001) and 72 h (*p* < 0.0001) after TBI in both DG and NG. When we compared the measurements at 24 h vs. 72 h after TBI, the rats from the DG demonstrated a statistically significant reduction in latency (*p* = 0.0334); however, we did not find differences in the NG (*p* = 0.9466). We then evaluated the effect of the cycle and found that the traumatized rats in the NG had a shorter latency than the traumatized rats in the DG (*F*_3.96_ = 77.08, *p* < 0.0001), a difference that was significant at 24 h after TBI (*p* = 0.0170) but not at 72 h after TBI (*p* = 0.8699).

We also quantified the number of errors (Fig. 3B). We found an influence of TBI within the groups with an increase 24 h after the trauma in both the DG (*p* = 0.0006) as in the NG (*p* = 0.00058) and at 72 h only for the DG (*p* = 0.0012) compared with their baselines. We also found an influence of the cycle, with the NG presenting fewer errors than the DG at 24 h (*p* = 0.0146) and 72 h (*p* = 0.0251) after TBI. However, 72 h after trauma, the group with TBI in DG showed a decrease in the frequency of mistakes compared with the 24 h post-TBI measurement (*p* = 0.0163) and baseline measurement (*p* = 0.0012). In contrast, 72 h after the trauma, there was no statistically significant difference in the NG compared with 24 h after TBI (*p* = 0.1515) or the baseline measurement (*p* = 0.0781).

### 3.3 Damage to the white matter presents histopathological variation depending on the time of TBI

**Fig. 4:**
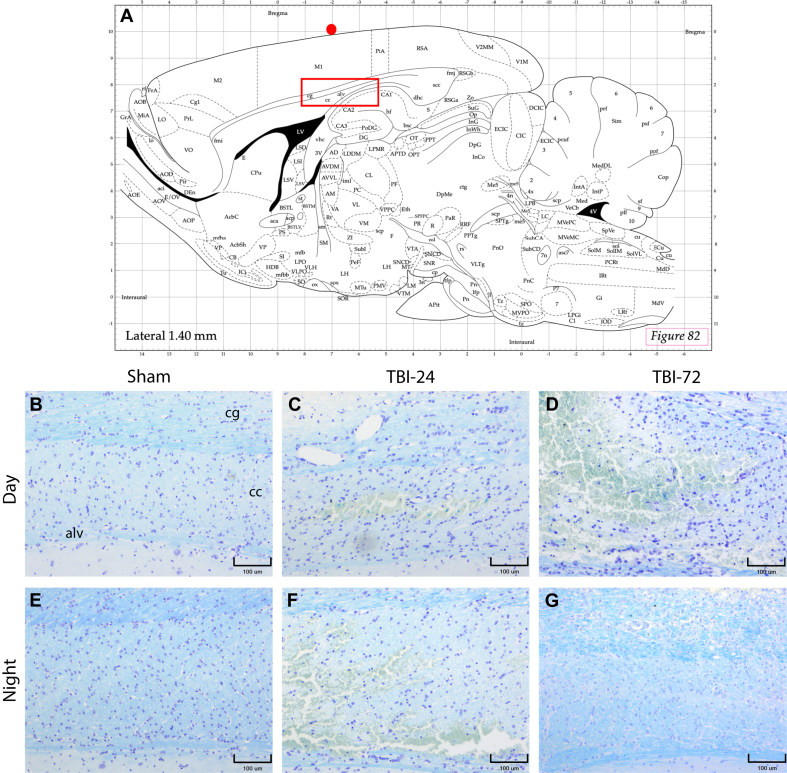
Rat brain regions of interest and WM-M structures analyzed after TBI. (A) Region of TBI (red dot) and area analyzed systematically throughout the work (highlighted red rectangle). Photomicrographs of the DG (B–D) and the NG (E–G). Bleeding in the CC area appeared to be lower at 24 h in the DG (C) than in the NG (F). However, at 72 h, the bleeding appears to be higher in the DG (D) than in the NG (G). (10 × ). cb, cingulum bundle; cc, corpus callosum; alv, hippocampal alveus. Bars = 100 μm. Klüver-Barrera stain method. (Atlas source: Paxinos, G. & Watson C. *The rat brain in stereotaxic coordinates: hard cover edition*. Access Online via Elsevier, 2006. Online tool by Matt Gaidica). (For interpretation of the references to colour in this figure legend, the reader is referred to the Web version of this article.)

To analyze the extent of damage in the WM-M that was neuroanatomically close to area of primary damage (Fig. 4), we performed a histopathological evaluation of a region that include the CC and CB (Fig. 4A) in the samples stained with Klüver-Barrera in the DG (Fig. 4B–D) and in the NG (Fig. 4E–G) in the Sham and 24 and 72 h after TBI. The histopathological description of each area can be found under its respective caption. Additionally, we analyzed the myelin damage scale of the samples.

**Fig. 5:**
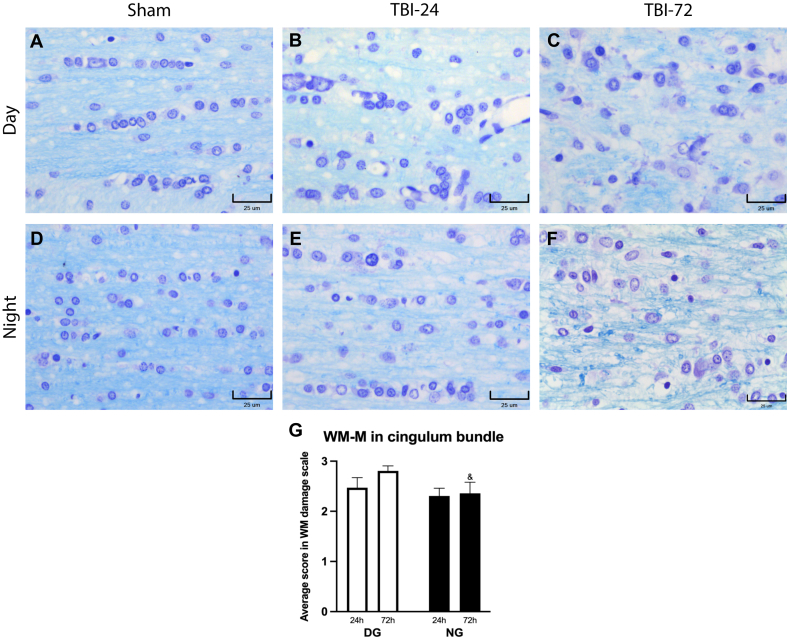
Rat cingulum bundle after TBI induction. Photomicrographs of CB of the Day (A–C) and Night (D–F) groups. In the Sham columns, both, the DG, and NG, showed a preserved cellular and fibrillar organization, in which the oligodendrocytes were observed with an interfascicular arrangement between the myelinated axons (within tracts of WM, oligodendrocytes are typically arranged in linear rows between the nerve fibers). Additionally, oligodendrocytes showed a preserved morphologic pattern. In the DG 24 h after the trauma (B) we observed considerable vasodilatation and disorganization of the oligodendrocytes, which involved loss of the interfascicular arrangement and disarrangement of myelinated fibers. Additionally, oligodendrocytes had an altered morphology with slight nuclear enlargement and vacuolization of the cytoplasm. These alterations were most marked at 72 h after trauma (C), when cell and myelin fiber organization were lost, and oligodendrocyte vacuolization was more evident. In contrast, the NG at 24 h after the trauma presented better preservation of WM-M (E), in which the presence of interfascicular oligodendrocyte arrangement was maintained, and there was little vasodilation, although there was a slight decrease in the intensity of myelin staining; also, there were no evident morphological changes in oligodendrocytes. At 72 h after the TBI (F), we began to observe disarrangement of the myelin fibers, an increase in vacuolization, reactive gliosis, and mild inflammatory infiltrate. (G) In both groups there was and increment of pyknotic nuclei, mainly 72 h after TBI. (G) The damage analysis showed greater average damage in the DG than in the NG, and this difference was statistically significant at 72 h after TBI. Data are expressed as the mean ± SEM. Kruskal–Wallis test and Mann–Whitney *U* test as *post-hoc*, ^&^*p* < 0.05 between DG and NG. Bars = 25 μm. Klüver-Barrera stain method.

The first area analyzed was the CB (Fig. 5). In summary, descriptively, the histopathology showed more significant damage in WM-M in the DG at 24 and 72 h after the trauma (Fig. 5A–C). The NG showed better preservation of WM-M at 24 and 72 h after trauma than the DG (Fig. 5D–F). The damage scale of the WM-M (Fig. 5G) did not show significant differences between 24 and 72 h after TBI in the DG (*p* = 0.1500) or the NG (*p* = 0.3000). When comparing the DG with the NG at 24 h, we did not find significant differences (*p* = 0.2000). However, when comparing the groups at 72 h post-TBI, there was a significant difference (*p* = 0.0500), with a lower score in the NG.

**Fig. 6:**
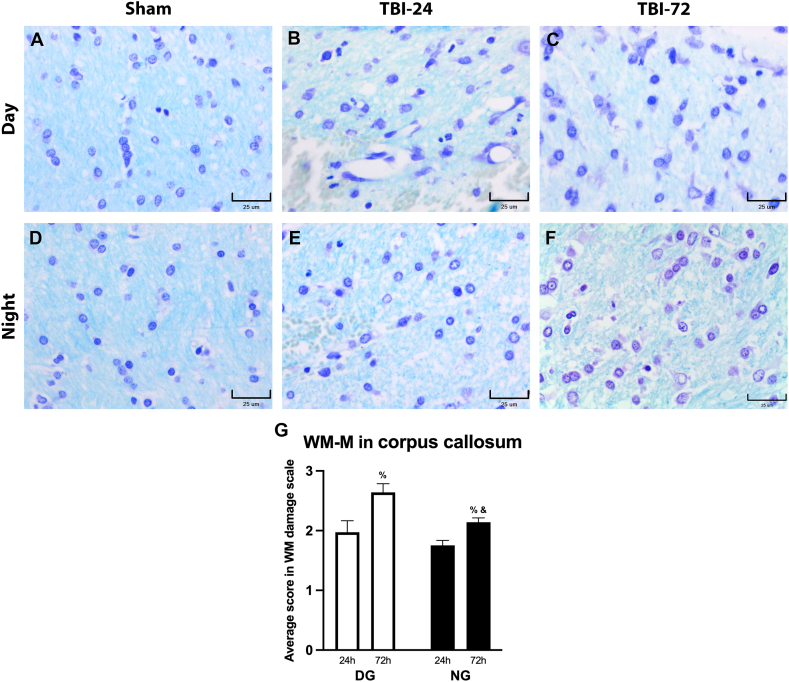
Rat corpus callosum after TBI induction. Photomicrographs of CC of the Day (A–C) and Night (D–F) groups. In the Sham columns, both the DG and NG, showed preserved cellular and fibrillar organization of WM-M and morphologically preserved cells. However, in the DG at 24 h after the trauma (B), we observed significant vasodilation, accentuated vacuolization of the oligodendrocytes near the area of hemorrhage, and disarrangement of oligodendrocytes and the myelin fibers. Karyopyknosis and karyorrhexis were noted at this time. At 72 h after the TBI (C), these changes were more accentuated with evident disorganization, vacuolization of the cells, and decrease of fibers; however, we did not observe vasodilation. In contrast, 24 h after the trauma (E), the NG presented an area of hemorrhage, and slight disarrangement of the fibers. The arrangement and morphology of the oligodendrocytes was preserved, although an increase in vacuolization was noticeable. Karyopyknosis and karyorrhexis were also noted at this time, but in lesser grade than in DG. At 72 h after the TBI (F), WM-M was better preserved, with some fascicular arrangement of oligodendrocytes. Cellular morphology changes were observed such as vacuolization, nuclear enlargement and evident nucleoli, reactive gliosis, and few pyknotic nuclei. The damage analysis (G) showed greater average damage in the DG, mainly at 72 h after the trauma. In the NG, the average damage was lower, with a significant difference at 72 h compared with the DG. Data are expressed as the mean ± SEM. Kruskal–Wallis test and Mann–Whitney *U* test as *post-hoc*, ^&^*p* < 0.05 between DG and NG; ^%^*p* < 0.05 different from 24 h after TBI. Bars = 25 μm. Klüver-Barrera stain method.

The second area analyzed was the CC (Fig. 6), in which we observed, descriptively, greater histopathological damage in the DG at both 24 h and 72 h after TBI (Fig. 6A–C). Compared with the DG, the NG presented characteristics consistent with better tissue preservation at both 24 h and at 72 h after TBI (Fig. 6D–F). The WM-M (Fig. 6G) showed a higher damage score between 24 and 72 h after TBI in the DG (*p* = 0.0500) and the NG (*p* = 0.0500). However, the comparison at 24 h between the DG with the NG was not significant (*p* = 0.3500). In contrast, at 72 h post-TBI, there was a statistically significant difference (*p* = 0.0500), with a lower score in the group with TBI at night.

### 3.4 The correlations in histopathology showed an influence of the diurnal variation on the damage

**Fig. 7:**
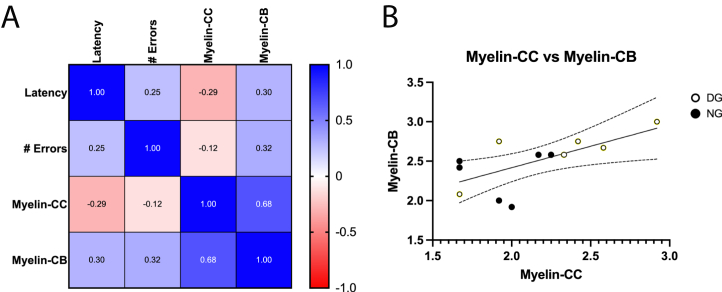
Correlation analysis of behavioral and histopathology variables. (A) Heatmap of Spearman correlation between the beam walk test and the average grade of myelin scale damage. (B) Simple linear regression with 95% confidence intervals of myelin scale between CB and CC. Empty circles correspond to day values, and full circles correspond to night values.

Finally, we analyzed whether the behavioral variables of latency and number of errors in the beam walk test were correlated with WM-M damage. Our results showed no significant correlations between histopathological and behavioral variables (Fig. 7A). However, the damage observed in the WM-M of the CB and CC was correlated. The linear regression between these variables was positive with a slope (m = 0.5451) that was significantly different from zero (*F*_1,10_ = 7.424, *p* = 0.0214), with a more significant correlation for day values (Fig. 7B).

## 4 Discussion

Numerous studies indicate the circadian onset or worsening of some disorders and that the time of day a pathological event occurs can influence the extent of the damage [27,28]. It is well documented that the timing onset of strokes and transient ischemic attacks most often occur in the early morning hours [29,30]. Various animal studies have also shown diurnal variations in the damage induced by focal or global ischemia, hemorrhagic stroke, TBI, and status epilepticus-mediated brain injury. This has been attributed to different neuroprotective mechanisms being activated or inhibited during the day [28].

According to our previous research, time of the day has an impact on damage recovery [12,13]. TBI recovery can be examined on multiple levels, from molecular to behavioral. Food consumption and weight loss, for example, as well as morphological changes in specific brain regions and whether the damage and recovery include mechanisms that modify neuronal myelin, can all be measured.

Our findings reveal that variables related to the individual's health (e.g., food intake and body weight), as well as other characteristics such as age and sex must be considered when examining what happens when a TBI occurs [[31], [32], [33]]. There is evidence that body weight can affect other the variables studied when TBI occurs. For example, in mice, higher basal body weight and change of body weight after TBI have been linked to more neuronal death and increased microglial and astrocyte activation in the damaged cerebral cortex [34]. In this context, the impact of diurnal variation on change in body weight from our data should be highlighted.

We showed that the NG had a smaller change in body weight than the DG after TBI. We hypothesized that these data were correlated with the change of food intake 24 h after TBI. At 24 h after TBI, we observed a significant difference in change in body weight between the DG and NG. The subjects of the DG had a greater loss in body weight. However, there were no differences in the change in food intake between the groups. Therefore, we attributed this finding to metabolic variables rather than the relation between food intake and body weight. For example, in critically ill patients, weight loss has been linked to a hypermetabolic state caused by various sudden metabolic changes induced by TBI, such as processes in the liver, pancreas, and kidney, resulting in a negative nitrogen balance, loss of lean tissue, lipolysis, and glucose intolerance with high insulin levels [35,36]. Within the first 24 h, hyperglycolysis is known to occur, with lipids serving as the predominant source of energy and proteins remaining stable until the end of the process [37]. Our data point to a greater weight loss in the first 24 h followed by a hypermetabolic state, which is more obvious in the TBI DG.

The results of the beam walk test are significant because we have previously described differences in damage between TBI that occurs during the day vs. the night [13]. In the present work, we confirmed these findings with a completely new test that evaluates different motor skills. Our aims were to see whether the results of the beam walk test might be related to the findings of WM-M histopathology and whether the diurnal variation in TBI had a role in any changes. Secondary damage activates pathophysiological pathways that result in sensorimotor deficits, behavioral problems, and long-term increased vulnerability to other physical and neurological disorders [38]. White matter is particularly significant because it primarily comprises connected myelinated axon tracts that allow more rapid neurotransmission between brain areas [9]. Damaged axons and oligodendrocytes may induce demyelination that spreads well beyond the initial injury site, resulting in sensory, cognitive, autonomic, and motor function impairments after TBI. In primary injury, traumatic demyelination of injured axons occurs at the lesion site, whereas subsequent processes can cause demyelination and deafferentation in areas remote from the initial insult [39].

In this regard, our analysis of the histopathological data demonstrated that the NG had less damage in both the CB and CC regions, particularly 72 h after TBI. These minor morphological changes in the NG could be explained by a diurnal variation-mediated neuroprotective effect, which would be regulated by local circumstances in both the CB and the CC. Neuroinflammation and excitotoxicity are two processes that could explain our histopathological findings and are related to these local conditions. First, pro- and anti-inflammatory interleukins have different release rates depending on the time of day [40,41]. Second, according to our previous findings, glutamate release and NMDA receptor expression, which are implicated in the neurotoxicity process, differ depending on the time of day [11,42]. Additionally, because axons are viscoelastic structures, they can resist significant stretching during daily activities [43]. However, with TBI, axons are thought to become fragile under dynamic mechanical loading, high strain rates, and fast brain deformation, predisposing them to physical damage [44]. However, the impact of diurnal variation on the WM-M after TBI has received little attention.

The CB is a large WM tract that connects subcortical nuclei to the cingulate gyrus and connects the frontal, parietal, and medial temporal lobes. Despite causing extensive anatomical disconnections, CB injuries usually result in very mild deficits, emphasizing the importance of parallel pathways and the distributed nature of the diverse activities of the CB. However, pain perception, spatial processing, spatial memory, and navigation alterations have only been observed in rodent models of CB lesions [45]. In human studies of CB alterations caused by TBI, only cognitive features [46], attention impairments [47], and memory deficits have been studied [48]. Few studies have focused on the significance of motor deficits. This is intriguing because CB has been shown to contribute to motor features [49], which have not been researched often in the murine model but could indicate a link to motor disruptions. However, it is worth noting that they are also subjected to diurnal variation following TBI.

Finally, the CC is a long strip of fibers that connects both cerebral hemispheres, and is responsible for the flow of interhemispheric information, which is why it represents a very important structure in the global functioning of the brain [50]. Damage to the CC causes a variety of distinct perceptual, motor, and cognitive impairments, ranging from coma to memory difficulty, including the so-called split-brain syndrome [51,52]. A prospective investigation of TBI-induced CC damage in humans indicated that 47% of individuals with TBI had significant CC lesions [53]. Another study discovered that 76.8% of TBI patients had severe CC loss [54]. Due to a hard attachment to the brain sickle (falx cerebri), a median sagittal fold of the dura, descending into the longitudinal cerebral fissure, and connections to the two independently mobile cerebral hemispheres, the CC is particularly vulnerable to injury [55].

For the first time, both WM-M regions (CB and CC) are histopathologically described after TBI, taking diurnal variation into account. Furthermore, we show that they are influenced by diurnal variation in neuroprotection. Finally, although other researchers have previously utilized the same scale to measure WM damage, we consider that it may be expanded to include more descriptive histological characteristics.

## 5 Conclusions

We show that diurnal variation is capable of modulating certain brain characteristics and processes that are involved in damage and neuroprotection following TBI. For example, rats subjected to TBI in the dark lost less body weight than those subjected to TBI in the light, with no change in food intake. Moreover, the rats subjected to TBI in the darkness had better performance in the beam walking test and presented less histological damage in the CC and the CB, as shown by the Klüver-Barrera staining. We need to do more studies to determine the mechanisms involved. We are interested in furthering the study of diurnal variation in oxidative stress, the immunological function of microglia and astrocytes, the levels of excitatory and inhibitory neurotransmitters, and the expression of their receptors. This could be the main factor that determines the balance between damage and neuroprotection. Moreover, this modulation could be reflected as a shift in the balance of damage/neuroprotection. Finally, the time of day when the injury occurs (in our case, a TBI) is data that should be used to evaluate the processes in the brain and develop better therapies.

## 6 Limitations and perspectives

Our work shows that the balance between damage and neuroprotection (evaluated with a beam walking test and histopathology of WM-M) after a TBI relates to diurnal variation. Our research group has focused on the influence of diurnal variation. We have found several data supporting more neuroprotection during the night, for example, NMDA receptor variation in the cortex [11] or histology in the gray matter of the motor cortex and hippocampus and with two different motor tests, a neurobehavioral scale and cylinder test [13]. However, our data must be seen in light of some limitations. Firstly, we consider the number of subjects used in these experiments based on obtaining a minimum sample size that would allow us to carry out an accurate statistical analysis and, considering the principle of the 3 R's. However, it will always be a limitation.

In this work, we have once again provided data that highlights that the time of day in which damage to the brain occurs is a factor to be considered. These could be relevant for the consideration of new neuroprotective strategies or therapies. However, our findings are only part of a more global panorama that we are trying to build with our research to provide a solid knowledge base about TBI. In this sense, our data must be considered in a framework that has several limitations: for example, our TBI model, although it has shown over several years to have high precision, is not without some uncertainty regarding the possible intra-investigator and intra-individual variations, we nevertheless consider that it yields outstanding data. Precise information on how a process of brain damage develops from a TBI.

Regarding food intake data and especially weight loss delta data, we consider it prudent to know which tissue, muscle or adipose, suffers the most significant loss and any water losses. In addition, laboratory metabolic measures such as serum glucose, lipids, insulin, and liver function tests will provide a complete overview of the metabolic variations between TBI during the day and the night. It is also possible that metabolic changes may be linked to corticosteroid variation [56]**;** thus, assessing its impact in future research would be relevant.

Finally, the focus is limited to a morphological description and analysis (specifically in WM-M of CG and CC) of the effects of TBI and to correlate them with our behavioral data. However, we are aware as a research group that our findings need to explain the differences we found. For instance, analyzing processes such as neurotransmitters and inflammatory and oxidative stress processes is required. Also, these findings could be supported by studies that directly detect diffuse axonal damage, such as MRI scans. With these limitations, our data provide an essential answer to our hypothesis.

Likewise, we would like to explore whether the time of the day in which a TBI occurred has an influence on many of the sequelae that appear, for example, memory deficit and anxiety/depression.

## Ethics statement

The animal study was reviewed and approved by the Faculty of Medicine Ethics Committee, Universidad Nacional Autónoma de México (UNAM) (project 018/2016; approved April 05, 2016).

## Funding

This project received support from PAPIIT: IN223417, IN228320 and IN228223, and CONACYT through the PhD fellowship 594 665.

## Author contribution statement

Ricardo Jesus Martinez-Tapia: Conceived and designed the experiments; Performed the experiments; Contributed reagents, materials, analysis tools or data; Wrote the paper. Francisco Estrada-Rojo: Performed the experiments; Wrote the paper. Teresita Guadalupe López-Aceves; Stephany García-Velasco; Veronica Rodríguez-Mata; Evelyn Pulido-Camarillo: Performed the experiments. Armando Pérez-Torres: Contributed reagents, materials, analysis tools or data. Erika Yazmín López-Flores; Perla E Ugalde-Muñiz; Roxana I Noriega-Navarro: Analyzed and interpreted the data. Luz Navarro: Conceived and designed the experiments; Contributed reagents, materials, analysis tools or data; Wrote the paper.

## Declaration of interest's statement

The authors declare no conflict of interest.

## Data availability statement

Data will be made available on request.
